# Promiscuous functions of the prion protein family

**DOI:** 10.3389/fcell.2015.00007

**Published:** 2015-02-10

**Authors:** Sophie Mouillet-Richard, Jean-Luc Vilotte

**Affiliations:** ^1^Toxicology, Pharmacology, and Cellular Signaling, Institut National de la Santé et de la Recherche Médicale Unité Mixtes de Recherche-S1124, Université Paris DescartesParis, France; ^2^Toxicology, Pharmacology, and Cellular Signaling, Sorbonne Paris Cité, Unité Mixtes de Recherche-S1124, Université Paris DescartesParis, France; ^3^Unité Mixtes de Recherche1313 Génétique Animale et Biologie Intégrative, Institut National de la Recherche AgronomiqueJouy-en-Josas, France

**Keywords:** prion protein, embryonic and fetal development, stem cells, aging, cell signaling, placenta, gonads, neuroprotection

From the discovery nearly 30 years ago of the cellular prion protein PrP^C^, the founder of the prion protein family, there has been a constant quest to dissect its biological function and that of its two homologs, Doppel and Shadoo. While clues were greatly anticipated from the generation of PrP null mice, alterations appeared quite imperceptible at first examination, beyond the clear-cut resistance to prion infection. Taking a closer look at these knockout mice, together with the generation of mice invalidated for Doppel and Shadoo has in the end yielded much information on the -sometimes overlapping- roles of these proteins. These in-depth investigations have also explored functions of the prion protein family beyond the central nervous system, which was obviously the first focus of interest since prion diseases are neurodegenerative disorders. This Frontiers Research topic on the promiscuous functions of the prion protein family incorporates contributions ranging from the field of developmental biology to that of structure-function, including aspects related to cell biology, signal transduction, and neuronal homeostasis.

Starting from the embryo, the contribution by Halliez et al. provides a comprehensive review of the impact of PrP invalidation on embryonic development, compiling data from both mice and zebrafish and highlighting the key cellular pathways affected by PrP^C^ deletion (Halliez et al., 2014). The review by Makzhami and colleagues is centered on the tissue with the second highest PrP^C^ expression after the brain, i.e., the placenta (Makzhami et al., 2014). It summarizes the recent data obtained with the help of PrP invalidated mice and discusses the pathophysiological implications stemming from the aberrant PrP^C^ expression in human gestational diseases. The review by Allais-Bonnet and Pailhoux is dedicated to the gonads, a unique tissue where PrP^C^, Shadoo and Doppel are all expressed, raising the question of a potential redundancy between the three proteins, as well as their roles in reproductive functions (Allais-Bonnet and Pailhoux, 2014). Mehrabian et al. provide a perspective on the potential relationship between PrP^C^ and the pathways involved in epithelial to mesenchymal transition, a process associated with major changes in cell adhesion properties and that physiologically takes place during embryonic development, while also involved in cancer metastasis (Mehrabian et al., 2014). A further connection with cancer is highlighted in the mini-review by Martin-Lannerée et al, which focuses on the contribution of PrP^C^ to stem cell biology and its recent association with tumor-initiating cells (Martin-Lanneree et al., 2014). At a cellular level, Sakudo and Onodera provide an overview of the data gathered by exploiting cell lines derived from PrP-null mice or constitutively knocked-down for PrP^C^, with special emphasis on the protective role exerted by this protein (Sakudo and Onodera, 2014).

Several contributions go down to the molecular scale and focus on the relationship between PrP^C^ and cell signaling. The mini-review by Roucou elaborates on the connection between PrP^C^ dimerization, proteolytic processing and the recruitment of cell signaling cascades (Roucou, 2014). Ochs and Málaga-Trillo provide a perspective on the recurrent link between PrP^C^-related signaling and src family kinases, in contexts ranging from embryonic cell adhesion to regulation of NMDA activity (Ochs and Malaga-Trillo, 2014). The protective function of PrP^C^ against NMDA-dependent excitotoxicity is the focus of the review by Black et al, which also discusses the pathophysiological implications of this regulation as to ischemic injury, neuroinflammation, and Alzheimer's disease (Black et al., 2014). This last issue relates to the identification of PrP^C^ as a cell-surface receptor for Abeta oligomers, and follow-up investigations on the contribution of PrP^C^ to Abeta toxicity, which is summarized in the review by Watt et al. (2014). This review additionally focuses on the contribution of PrP^C^ to zinc homeostasis, and discusses how age-regulated deregulation of the interplay between PrP^C^, lipid rafts and zinc may contribute to Alzheimer's disease. The fate of PrP^C^ during aging is further discussed in the review by Gasperini and Legname, which notably highlights the changes in the biochemical properties and lipid raft association of PrP^C^ in aged animals (Gasperini and Legname, 2014).

Further zooming on the molecule itself, the review by Rezaei provides a global view of the biochemical and structural similarities between PrP^C^, Doppel and Shadoo, as well as their specificities, in relation with their propensity to misfold (Rezaei, 2015).

Collectively, these works underscore the advance in our understanding of the functions exerted by the prion protein family and underlines their versatile roles according to the cellular context and interacting partners involved. Finally, they provide some future directions for further dissecting how the deregulation of these proteins functions can cause or contribute to pathological conditions.

## Conflict of interest statement

The authors declare that the research was conducted in the absence of any commercial or financial relationships that could be construed as a potential conflict of interest.
